# Comparison of Whole-Genome Sequences from Two Colony Morphovars of *Burkholderia pseudomallei*

**DOI:** 10.1128/genomeA.01194-15

**Published:** 2015-10-15

**Authors:** Pei-Tan Hsueh, Yao-Shen Chen, Hsi-Hsu Lin, Pei-Ju Liu, Wen-Fan Ni, Mei-Chun Liu, Ya-Lei Chen

**Affiliations:** a Department of Biological Science, National Sun Yat-sen University, Kaohsiung, Taiwan; b Division of Infectious Diseases, Kaohsiung Veterans General Hospital, Kaohsiung, Taiwan; c Department of Internal Medicine, National Yang-Ming University, Taipei, Taiwan; d Section of Infectious Disease, Department of Medicine, E-Da Hospital, Kaohsiung, Taiwan; e Department of Biotechnology, National Kaohsiung Normal University, Kaohsiung, Taiwan

## Abstract

The entire genomes of two isogenic morphovars (vgh16W and vgh16R) of *Burkholderia pseudomallei* were sequenced. A comparison of the sequences from both strains indicates that they show 99.99% identity, are composed of 22 tandem repeated sequences with <100 bp of indels, and have 199 single-base variants.

## GENOME ANNOUNCEMENT

*Burkholderia pseudomallei* is a soil-borne pathogen that causes community-acquired and life-threatening melioidosis, an endemic disease that occurs in Southeast Asia and northern Australia (1). This bacterium exhibits the unusual trait of changeable colony morphology on Ashdown’s selective media after incubation (2, 3). Different colony morphovars stemming from isogenic strains have been demonstrated to exhibit distinct proteomic profiles and pathogenic patterns in animals (4, 5). We have previously demonstrated that isogenic *B. pseudomallei* strains that formed wrinkled and dry colonies were more virulent in animals than those with a smooth and mucoid colony morphology (5). To gain insight into the genomic structures of different morphovars, the entire genomic sequences of both strains were sequenced and compared.

*B. pseudomallei* vgh16 (synonym, vgh19) was obtained from the blood of a melioidosis patient with septicemia. Typically, pink, wrinkled, and dry colonies (i.e., vgh16W) as well as red, smooth, and mucoid colonies (i.e., vgh16R) can be derived from the parental strain *B. pseudomallei* vgh16 cultured on Ashdown’s media. A single colony of each morphovar was picked and the total DNA extracted using a mini-QIAamp DNA isolation kit (Qiagen, Germany). The entire genomic sequence was determined using next-generation sequencing with PacBio (Pacific Bioscience of California, Inc., Menlo Park, CA, USA) technologies. By generating insert target continuous long reads averaging 20 kb, read processing and *de novo* assembly were performed using the HGAP program (version 3; bioinformatics analysis served by Yourgene Biotech, Inc., New Taipei City, Taiwan). The entire genomic sequences of the two isogenic strains were compared using the MUMmer program (version 3.22). Tandem repeated sequences in the chromosomes were analyzed using mreps software (http://mreps.univ-mlv.fr/).

Both strains had a large chromosome (4,038,845 bp, vgh16W; 4,038,504 bp, vgh16R) and a small chromosome (3,227,965 bp, vgh16W; 3,228,004 bp, vgh16R). The nucleotide sequences had 99.99% identity. No DNA rearrangements were found. There were 22 sites of tandem repeated sequences with <100 bp of indels (*n* = 7, chromosome 1; *n* = 15, chromosome 2). Variations in 199 bases were noted to insertions, deletions, or polymorphisms.

### Nucleotide sequence accession numbers.

The whole-genome sequences of *B. pseudomallei* vgh16W and vgh16R have been deposited at GenBank under the accession numbers CP012517 (vgh16W, chromosome 1), CP012518 (vgh16W, chromosome 2), CP012515 (vgh16R, chromosome 1), and CP012516 (vgh16R, chromosome 2).
